# Integrating New Approach Methodologies to Address Environmental Pancreatic Toxicity and Metabolic Disorders

**DOI:** 10.3390/biology14010085

**Published:** 2025-01-17

**Authors:** Yue Ge

**Affiliations:** Center for Computational Toxicology and Exposure, US Environmental Protection Agency, Research Triangle Park, NC 27711, USA; ge.yue@epa.gov

**Keywords:** NAMs, environmental chemical, dietary factor, pancreatic toxicity, metabolic disorder, commentary

## Abstract

Advancing our understanding of pancreatic toxicity and metabolic disorders caused by environmental exposures requires innovative approaches. The pancreas, a vital organ for glucose regulation, is increasingly recognized as a target of harm from environmental chemicals and dietary factors. Traditional toxicological methods, while foundational, often fail to address the mechanistic complexities of pancreatic dysfunction, particularly under real-world conditions involving multiple exposures. New Approach Methodologies (NAMs)—including high-throughput screening (HTS), OMICS technologies, computational modeling, and advanced in vitro systems—offer transformative tools to tackle these challenges. NAMs enable the identification of mechanistic pathways, improve testing efficiency, and reduce reliance on animal testing. This commentary explores the integration of NAMs into pancreatic toxicity screening, addresses critical gaps in evaluating the cumulative risks of chemical and dietary exposures, and proposes solutions for integrating the pancreas into toxicity screening through NAMs. By highlighting recent advancements and emphasizing their adoption in environmental toxicity assessment frameworks, this work demonstrates the potential of NAMs to revolutionize environmental health research, inspire interdisciplinary collaboration, and protect public health.

## Simple Summary

Environmental chemicals and dietary factors can harm the pancreas, an organ essential for regulating blood sugar and energy balance. Traditional methods to study these effects have limitations, prompting the development of advanced tools known as New Approach Methodologies (NAMs). These include high-throughput screening (HTS), OMICS technologies, computational modeling, and in vitro models. NAMs provide critical insights into how chemicals impair insulin production and disrupt glucose metabolism, contributing to conditions like diabetes and metabolic disorders. This commentary emphasizes how harmful chemicals and unhealthy diets, individually and in combination, worsen metabolic health. By integrating NAMs into chemical risk assessments, researchers can better predict and mitigate the impacts of environmental exposures, paving the way for improved prevention and treatment strategies while reducing the burden of metabolic diseases.

## 1. Introduction

The pancreas is a critical organ for maintaining metabolic homeostasis, playing a central role in regulating glucose levels and energy balance [1]. Through its endocrine functions, it secretes insulin and glucagon, which coordinate glucose uptake and release, ensuring metabolic stability [1]. Disruptions in these functions can lead to metabolic disorders such as type 2 diabetes, obesity, cardiovascular disease, and cancer [2], which have become increasingly prevalent globally. Environmental chemicals and dietary factors are key contributors to pancreatic dysfunction [3]. Exposure to chemicals like bisphenol A (BPA) [4], arsenic [5], and phthalates [6] has been shown to impair insulin secretion, promote insulin resistance, and disrupt glucose metabolism. For example, BPA interferes with insulin signaling and glucose regulation [4], while arsenic exposure damages β-cell function, leading to impaired glucose regulation [5]. High-fat diets (HFDs) exacerbate these effects, amplifying the risks of metabolic disorders [7,8]. These findings highlight the importance of studying individual chemicals and dietary factors, as well as their combined effects, to fully understand their role in pancreatic dysfunction.

Traditional toxicological approaches often face limitations in addressing both individual and combined exposures. These methods are time-intensive, costly, and often lack the capacity to capture the mechanistic complexities of pancreatic toxicity. New Approach Methodologies (NAMs) have emerged as a promising alternative, enabling more efficient and mechanistically informed evaluations of chemical and dietary factors [9,10]. By focusing on bioactivity endpoints and pathway-based data, NAMs provide critical tools for modernizing pancreatic toxicity screening and improving risk assessments. This commentary explores the current state of knowledge on pancreatic toxicity and discusses how NAMs and advanced methodologies can address these gaps, ultimately advancing our ability to assess and manage pancreatic health risks.

## 2. Pancreas as a Target of Environmental Chemicals

The pancreas has long been recognized as a target for chemical-induced toxicity, with early evidence emerging in the 1940s when alloxan, a glucose analogue, was found to induce type 1 diabetes in rabbits by destroying insulin-producing cells [11]. Occupational exposure to vinyl chloride (VC) was first associated with hepatic hemangiosarcoma in workers at the B.F. Goodrich Plant in 1974 [12]. A subsequent study identified a 4- to 5-fold increased incidence of hepatic and pancreatic tumors in workers employed at a Swedish VC manufacturing plant [13], a foundational study that identified increased incidences of pancreatic tumors associated with chemical exposure.

Table 1 summarizes 10 representative studies on the effects of chemicals and/or their mixtures on the pancreas, focusing on research conducted between 1976 and 2018. These studies highlighted adverse outcomes such as disrupted insulin secretion [14,15], tumor development [11,12], and impaired glucose metabolism [16,17], which are valuable for understanding the mechanisms of pancreatic toxicity induced by well-known and extensively characterized environmental chemicals or pollutants across different categories. Traditional toxicological approaches dominated this period, utilizing in vivo animal models and epidemiological data to assess the effects of chemicals on pancreatic function. These methods offered holistic perspectives on pancreatic toxicity by capturing whole-organism interactions and real-life exposure scenarios. However, a progressive shift toward NAMs began in the late 2000s and early 2010s and provided mechanistic insights into toxicity through in vitro bioactivity-based assays, such as HTS, OMICS, and computational models [9,10], addressing limitations of traditional methods and growing complexity of environmental exposures. The growing complexity of environmental exposures including chemical mixtures, chronic low-dose exposures, and cumulative effects, further underscored the need for integrating NAMs into pancreatic toxicity research. This transition reflects a critical evolution in pancreatic toxicology, where traditional approaches and NAMs began to complement each other. This integrated perspective is essential for addressing gaps in understanding how environmental exposures contribute to pancreatic dysfunction and metabolic diseases.

**Table 1:** Summarizes 10 key studies on the effects of environmental chemicals on pancreas-mediated metabolic syndromes and diseases from 1976 to 2018.

Chemical	Outcome	Approach	Model	Year	Citations
Alloxan	Insulin-producing β-cells	Traditional toxicology	In vivo	2008	[11]
VC	Tumor incidence	Epidemiology	Human	1976	[13]
BPA	β-cell function	Traditional toxicology	In vivo	2012	[4]
HFD	Metabolic pathways	Traditional toxicology	In vivo	2010	[7]
Arsenic	Insulin secretion	Traditional Toxicology	In vivo	2016	[5]
Phthalates	Insulin resistance	Epidemiology	Human	2016	[6]
DEHP	Insulin homeostasis	Traditional toxicology	In vivo	2011	[14]
PCBs	Glucose metabolism	Epidemiology	Human	2014	[15]
Dioxins	Insulin secretion	Epidemiology	Human	2008	[16]
PAHs	Glucose homeostasis	Epidemiology	Human	2015	[17]

## 3. Integrated Modern Methodologies Provide Mechanistic Insights into Pancreatic Toxicity

Integrated modern methodologies encompass novel methodologies, such as NAMs, and advanced techniques, such as HTS, OMICS technologies, computational modeling, and in vitro human pluripotent stem cell (hPSC) system. These approaches and their integrations provide deeper mechanistic insights into chemical-induced pancreatic toxicity, reduce reliance on traditional animal testing, improve efficiency, and address the complexities of environmental exposures [18,19,20]. By integrating these methodologies, toxicologists can better understand the intricate pathways and processes involved in environmental chemical-induced pancreatic dysfunction, particularly in metabolic pathways linked to β-cell function and insulin regulation. For instance, advanced HTS techniques have been employed to identify small molecules that enhance β-cell function and viability under metabolic stress, offering potential therapeutic interventions for diabetes and metabolic disorders [18]. Automated high-content imaging has been used for whole pancreatic islets’ responses to environmental stressors [19]. As for OMICS, Lytrivi et al. (2020) combined proteomics and transcriptomics to identify critical drivers and pathways of the β-cell lipotoxic response for the first time and identified novel therapeutic targets for type 2 diabetes [20]. Single-cell transcriptomics was also applied to analyze mouse β and α cells at the single-cell level, providing insights into the mechanisms of cellular heterogeneity, cell number expansion, and maturation of both β and α cells [21]. Additionally, multi-OMICS, including metabolomics, transcriptomics, and proteomics, were integrated to investigate tumor metabolism in collected samples from pancreatic ductal adenocarcinoma (PAAD) patients. The study provided new insights into prognosis prediction and therapy for pancreatic ductal adenocarcinoma patients, emphasizing the interconnected nature of these high-throughput approaches [22].

Table 2 summarizes representative studies conducted primarily between 2018 and 2024 that utilize NAMs to investigate pancreatic toxicity. These studies were selected based on their sophisticated application of HTS and OMICS technologies, particularly those focusing on the disruption of metabolic pathways and their integration with advanced in vitro models. The studies highlight the impact of environmental exposures on β-cell function, metabolic disruptions, and adverse health outcomes. In addition to HTS and OMICS, computational models play a critical role in predicting chemical effects on pancreatic β-cell function [23,24]. For instance, multi-scale computational and statistical models have integrated epidemiology data to predict metabolic syndrome risk in children with prenatal exposure to environmental chemical mixtures [23]. Machine learning and data mining methods were implemented to understand environmental exposures in diabetes etiology, offering a powerful platform for toxicity and disease prediction [24].

Advanced in vitro models, including rodent- and human-derived β-cells, have enabled detailed investigations into chemical toxicity and its effects on β-cell function [25,26]. These models are pivotal for studying insulin production and viability, offering insights into toxicant-induced metabolic syndromes. A major advancement is the use of hPSC cultures, which overcome many limitations of traditional β-cell models [26]. hPSC-derived β-like cells closely mimic the function of human pancreatic β-cells, providing a physiologically relevant platform for assessing chemical toxicity [26]. These cells are also glucose-responsive and insulin-secreting, making them suitable for testing pancreatic function across diverse exposures. In addition to the advanced hPSC cell culture system, co-culture models have further advanced our understanding of how metabolism is regulated by the endocrine system, including the pancreas, liver, adipose tissue, thyroid, adrenal glands, and pituitary. For instance, studies on the development of a pancreas–liver organ-on-chip coculture model for investigating organ-to-organ interactions illustrated the potential of organ-on-chip technology for reproducing crosstalk between the liver and pancreas [27,28]. To define the mechanisms and toxicities of suspected environmental chemicals, it is essential to explore their impacts on multiple endpoints across various components of the endocrine system.

In summary, the integration of HTS, OMICS technologies, computational modeling, and advanced in vitro models provides a robust framework for investigating chemical-induced pancreatic toxicity. These methodologies, as part of NAMs, enhance testing efficiency and uncover mechanistic insights into β-cell dysfunction. Recent studies have particularly emphasized the interplay between environmental exposures and metabolic disruptions, highlighting the importance of innovative approaches to address these complex challenges.

## 4. Interplay Between Environmental Chemicals and Dietary Factors on Metabolic Dysfunction

HFD is well-established as a major contributor to metabolic dysfunction and is considered a key driver of metabolic syndrome [29]. Emerging evidence indicates that environmental exposures to metabolically disrupting chemicals can exacerbate the adverse effects of an HFD [7,8]. For example, exposure to polycyclic aromatic hydrocarbons (PAHs) has been shown to lead to obesity, insulin resistance, and inflammation, particularly when followed by an HFD in adulthood, and the lipophilic nature of compounds such as PAHs allows them to accumulate at higher concentrations or exhibit prolonged half-lives under HFD conditions, potentially amplifying their adverse metabolic effects [30]. A recent proteomic study [31] compared the pancreatic proteome of mice exposed to vinyl chloride (VC) and an HFD, both individually and in combination, with that of normal mice fed a low-fat diet (LFD). The study aimed to identify differentially expressed cytokines, enzymes, and phosphorylated AKT kinases, focusing on proteins integral to pancreatic-mediated metabolic dysfunction. Notably, the combination of VC and HFD resulted in amplified metabolic disruptions compared to individual exposures, including significant alterations in cytokine and enzyme profiles. These findings highlight a synergistic interaction in which combined exposures exacerbate the severity of pancreatic dysfunction and metabolic syndrome. By quantitatively measuring and comparing proteins, the study provided mechanistic insights into the roles of altered proteins in the pathogenesis of metabolic syndrome induced by VC and HFD. These results emphasize the need for further investigations into how environmental and dietary factors interact to impact metabolic health, particularly through their cumulative effects on pancreatic function.

**Table 2:** List of 11 studies using NAMs and in vitro cell systems for enhancing the understanding of how environmental chemicals impact pancreatic function, toxicity, and adverse health outcomes.

Chemical/Cells	Outcome	Approach	Model	Year	Citation
Multiple Chemicals	β-cell function	HTS	In vitro	2021	[18]
Multiple Chemicals	β-cell function	HTS	In vivo	2023	[19]
Multiple Chemicals	β-cell function	Proteomics/Transcriptomics	In vitro	2020	[20]
Cells	β and α cell development	Single Cell Transcriptomics	In vitro	2017	[21]
Patient Tissues	Pancreatic cancer	OMICS	In vitro	2023	[22]
Chemical Mixtures	Diabetes	Computational Model	In vivo	2024	[23]
Multiple Chemicals	Pancreatic β-cell function	Machine Learning	In vitro	2023	[24]
Cells	Multi-metabolic outcomes	hPSC	In vitro	2021	[25]
Cells	Interactive effects	Cell Co-Cultures	In vitro	2020	[27]
Cells	Interactive effects	Org Chip	In vitro	2020	[28]
VC + HFD	Metabolic disorder	Proteomics	In vivo	2023	[31]

## 5. Conclusions

Environmental pancreatic toxicity represents a critical intersection between environmental exposures and metabolic health, with far-reaching implications for public health and regulatory science. This commentary highlights the transformative role of New Approach Methodologies (NAMs) in addressing the complexities of pancreatic toxicity and associated metabolic disorders. Advanced methodologies, such as high-throughput screening (HTS), OMICS technologies, computational modeling, and in vitro systems, provide unparalleled insights into disrupted metabolic pathways and mechanistic processes. These tools not only enhance testing efficiency but also uncover the intricate interplay between environmental exposures and metabolic dysfunction, bridging gaps left by traditional approaches. Recent advancements further underscore the promise of NAMs. For example, the NEMESIS project identified novel biomarkers and methodologies for detecting and understanding metabolic disruptors, offering actionable insights into adverse effects and their mechanisms [32]. System toxicology frameworks [33] provide predictive tools to address complex hazard assessments, demonstrating how integrative approaches can revolutionize toxicological research. Additionally, strategies for overcoming barriers to NAM adoption [34] are pivotal for accelerating the implementation of these advanced methodologies into regulatory frameworks. Future research should prioritize expanding the application of NAMs to diverse metabolic endpoints, including those influenced by combined exposures to environmental chemicals and dietary factors. By integrating molecular, computational, and systemic approaches, researchers can enhance predictive capabilities and address the growing challenges posed by metabolic disorders in real-world scenarios. Interdisciplinary collaboration and innovation will be essential for refining our understanding of environmental pancreatic toxicity and developing actionable strategies to mitigate its impacts on human health.
